# Revolutionizing Blood Collection: Innovations, Applications, and the Potential of Microsampling Technologies for Monitoring Metabolites and Lipids

**DOI:** 10.3390/metabo14010046

**Published:** 2024-01-11

**Authors:** Eleonora Bossi, Elena Limo, Lisa Pagani, Nicole Monza, Simone Serrao, Vanna Denti, Giuseppe Astarita, Giuseppe Paglia

**Affiliations:** 1 Department of Medicine and Surgery, Proteomics and Metabolomics Unit, University of Milano-Bicocca, 20854 Vedano al Lambro, Italy; e.bossi13@campus.unimib.it (E.B.); elena.limo@unimib.it (E.L.); lisa.pagani@unimib.it (L.P.); n.monza@campus.unimib.it (N.M.); vanna.denti@unimib.it (V.D.); 2 Department of Biochemistry and Molecular & Cellular Biology, Georgetown University, Washington, DC 20057, USA; gastarita@gmail.com

**Keywords:** metabolomics, lipidomics, dried blood spot, microsampling, drug discovery, personalized medicine

## Abstract

Blood serves as the primary global biological matrix for health surveillance, disease diagnosis, and response to drug treatment, holding significant promise for personalized medicine. The diverse array of lipids and metabolites in the blood provides a snapshot of both physiological and pathological processes, with many routinely monitored during conventional wellness checks. The conventional method involves intravenous blood collection, extracting a few milliliters via venipuncture, a technique limited to clinical settings due to its dependence on trained personnel. Microsampling methods have evolved to be less invasive (collecting ≤150 µL of capillary blood), user-friendly (enabling self-collection), and suitable for remote collection in longitudinal studies. Dried blood spot (DBS), a pioneering microsampling technique, dominates clinical and research domains. Recent advancements in device technology address critical limitations of classical DBS, specifically variations in hematocrit and volume. This review presents a comprehensive overview of state-of-the-art microsampling devices, emphasizing their applications and potential for monitoring metabolites and lipids in blood. The scope extends to diverse areas, encompassing population studies, nutritional investigations, drug discovery, sports medicine, and multi-omics research.

## 1. Introduction

Blood serves as a crucial biological fluid in a myriad of applications, spanning research, health, diagnosis and drug monitoring. The conventional method of venous blood sampling, exceeding 1 mL, is regarded as the gold standard but entails drawbacks like invasiveness, the necessity for a sterile environment, and the involvement of trained phlebotomists [1]. Moreover, it poses potential risks such as exposure to pathogens, demanding intricate sample preparation procedures, and precise shipping conditions, resulting in elevated costs [2]. As the landscape shifts towards personalized medicine and broadened population studies, necessitating sample size reduction, the need for innovative blood collection tools becomes imperative [3].

Blood microsampling, involving small volumes (≤150 μL) of capillary blood typically derived from the fingertip with a blood lance, has garnered attention for its simplicity, rapidity, minimal invasiveness, and user-friendly nature. 

Recently, this type of blood collection has gained increasing interest for several reasons: first, it is a simple, rapid, minimally invasive and user-friendly method of blood sampling that does not require trained personnel and that can be performed at home as a point-of-care test [4]. Blood microsampling, which consists of dried blood, does not require any centrifugation or aliquoting, requires easier protocols, and is easily stored and transported since it requires less stringent shipping conditions, involving lower costs [1]. 

Apart from its logistical advantages, blood microsampling presents a viable alternative to venous blood sampling, fostering a shift towards personalized, subject-centered approaches. Its versatility extends to automated workflows, mitigating biological risks by inactivating pathogens within the dried matrix. 

Moreover, microsampling allows for self-sampling procedures and can be implemented in remote areas, enhancing healthcare accessibility [5]. Furthermore, blood microsampling facilitates longitudinal studies through multiple sampling opportunities, catering to diverse settings.

The compatibility of blood microsampling with various bioanalytical techniques, including mass spectrometry (MS), and enzyme immunoassays [6], has spurred increased research activity. Applications range from diagnostics and large-scale screenings in clinical studies to drug testing [7,8,9,10,11,12,13,14,15,16], forensic toxicology [17], and sports-related analyses [18]. The integration of MS-based analysis has opened avenues in omics sciences, including metabolomics [19], proteomics [20], and lipidomics [21]. This makes blood microsampling a promising tool for overcoming challenges associated with traditional blood sampling in omics studies, addressing issues of invasiveness, and facilitating remote sampling and longitudinal studies.

In this review, we will provide a comprehensive overview of recent innovations in commercially available microsampling devices, focusing on their applications, with particular emphasis on their potential for monitoring metabolites and lipids in blood.

## 2. Microsampling Devices

**Figure 1:**
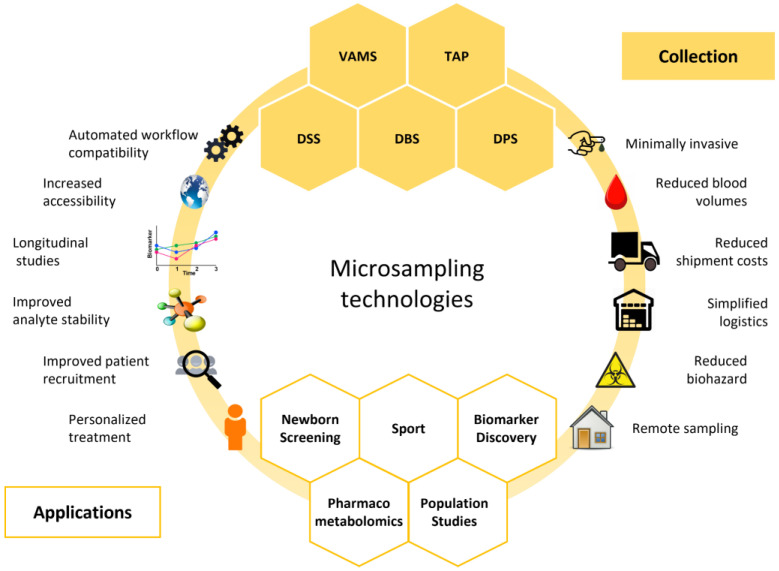
Graphical summary of microsampling technologies. Blood microsampling has various applications and offers several advantages compared to traditional venous blood sampling. DBS: dried blood spot; DPS: dried plasma spots; DSS: dried serum spots; TAP: touch-activated phlebotomy; VAMS: volumetric absorptive microsampling.

Presently, several commercially available microsampling devices cater to the diverse needs of these applications (Figure 1 and Table 1).

### 2.1. Dried Blood Spot (DBS)

DBS, the inaugural microsampling technique dating back to 1963, was initially developed for large-scale newborn screening of phenylketonuria [22]. As the first and most extensively utilized microsampling method, it involves collecting a few drops of blood on paper-based sampling cards obtained by pricking the subject’s finger with a blood lancet. These samples are then dried, transported, and stored until analysis is conducted. Notably, DBS captures whole blood samples, encompassing both intracellular and extracellular components, thereby offering diverse opportunities for clinical research [23].

The contemporary landscape boasts various types of DBS devices catering to evolving needs.

#### 2.1.1. DBS on Filter Paper

**Figure 2:**
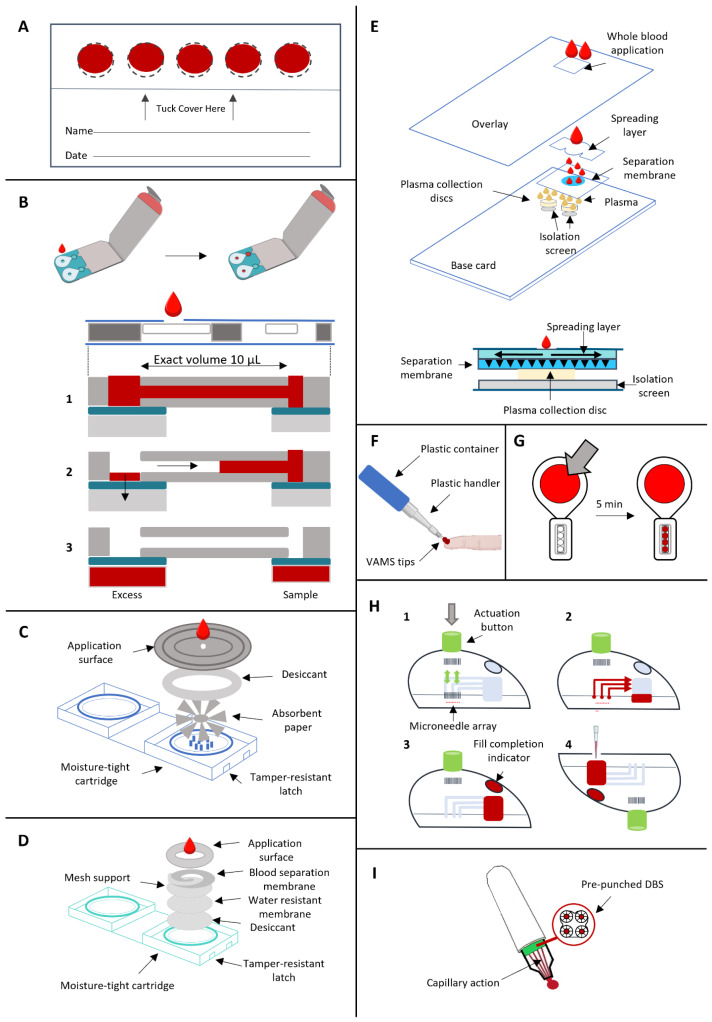
Microsampling devices. (**A**) DBS sample cards. (**B**) Capitainer^®^ B (Capitainer AB, Stockholm, Sweden) (qDBS) with cross-sectional view. It involves a microfluidic system that allows the collection of an exact volume of 10 µL of whole blood. 1B—a single drop of blood is collected and fills the capillary microchannel; 2B—the thin film located at the inlet dissolves and the blood in excess is absorbed by a paper substrate; 3B—the thin film at the outlet is dissolved, the capillary channel is emptied thanks to capillary forces, and 10 µL of blood is absorbed on a pre-cut paper disk. (**C**) HemaSpot^TM^ (Spot On Sciences, San Francisco, CA, USA) HF cross-sectional view for the quantitative collection of whole blood. (**D**) HemaSpot^TM^ (Spot On Sciences, San Francisco, CA, USA) SE cross-sectional view. It allows the serum separation from whole blood by lateral flow. (**E**) Telimmune DUO (Telimmune, West Lafayette, IN, USA) cross-sectional view for the plasma separation from whole blood without centrifugation. (**F**) Mitra^®^ device (Trajan Scientific and Medical, Melbourne, VIC, Australia) using VAMS tips to collect whole blood. (**G**) TASSO (Tasso Inc., Seattle, WA, USA) M-20 for whole blood collection. (**H**) TAP device (YourBio Health, Medford, MA, USA) to collect capillary blood samples by deploying and withdrawing a microneedle array, generating a vacuum pressure that allows the collection. 1H—the button at the top of the device is pushed and the microneedle array is deployed and then withdrawn; 2H—the vacuum draws blood into the sample container. 3H—the visual indicator turns red when the collection is completed. 4H—The device is removed and sampling is completed (**I**) hemaPEN^®^ (Trajan Scientific and Medical, Melbourne, VIC, Australia) for the collection of whole blood on 4 pre-punched DBS through capillary action.

The Whatman^®^ 903 Protein Saver Card (Cytiva, Global) serves as a paper-based substrate for collecting blood volumes (approximately 75–80 μL per spot) on a fixed circular surface with a diameter of 1.27 cm (Figure 2A) [24]. It is the most used filter paper but it is not the only commercially available one. Following collection, these sample cards are air-dried at room temperature for a minimum of 3 h and then transported for subsequent analysis, with stable storage even when sealed in their envelopes for mailing to laboratories [25]. For precise measurements, fixed-diameter spots (usually 3 or 6 mm) are punched out of the paper substrate, and an extraction protocol is executed using appropriate buffers.

However, a notable challenge in DBS methodology lies in variability, impacting analyte quantification due to fluctuations associated with hematocrit (HCT). HCT, representing the ratio of red blood cells to total blood volume, fluctuates between 36 and 50% based on factors such as race, sex, age, fluid intake, and overall health [25]. When blood is collected on the sample card, HCT influences spot size, homogeneity, and extraction recovery. Higher HCT values result in increased blood viscosity, affecting sample flow and distribution through the paper substrate, adversely impacting reproducibility. In essence, higher HCT leads to smaller spots on the sample card, creating an inverse relationship between HCT and spot size [26,27].

The use of fixed-diameter punches, while convenient, introduces unreliability in volumes, limiting the accuracy of analyte quantification. Moreover, changes in HCT values impact the total analyte amount and extraction recovery rate. Higher HCT levels lead to lower extraction recoveries, hindering the extraction process in DBS, ultimately causing underestimation of analytes [28]. Analytical errors also stem from DBS components, including the paper substrate, influencing maximum loading capacity, blood spreading, analyte stability, and recovery. Factors like storage conditions, shipping temperature, humidity, and environmental conditions during sample collection or spotting contribute to variable analyte distribution. Temperature and humidity variations, in particular, can alter spot size and lead to uneven distribution of analytes within the spot [28].

Numerous attempts have been made to address the HCT-related issues in DBS. To mitigate the variability resulting from a fixed diameter spot, Velghe and Stove propose a volumetric application of a fixed amount of blood coupled with whole spot analysis. Specific devices designed for volumetric dried blood sampling aim to retain the advantages of DBS while resolving these challenges [29]. These quantitative microsampling devices provide a robust method to overcome the bias associated with HCT spot size, but they are not able to eliminate the negative effects of HCT on extraction efficiency. Therefore, it is necessary to optimize extraction protocols and assess analyte recoveries at different HCT values to minimize this bias and ensure that it is within acceptable bounds [1]. 

Typically, an internal standard (IS; stable isotopically labeled target metabolite) is added during extraction to correct for variation associated with extraction and recovery. In practice, the variation in the HCT can lead to a recovery bias that only affects the analyte and cannot be corrected by the addition of the IS (the analyte/IS ratio is fixed and the IS is not affected by the recovery bias). Co-spiking of blood with IS together with analyte and IS co-extraction has been proposed as a way to overcome the recovery bias by nullifying the effect of HCT-based recovery bias. However, co-spiking blood with IS prior to substrate application is not feasible for DBS workflows. Abu-Rabie et al. investigated a method based on IS spray addition prior to extraction protocol applied to drug discovery and development. According to this study, HCT-based recovery bias was more significant in tests with lower recovery rates (40–60% absolute recovery), while tests with higher recovery rates (>90%) were not significantly affected by this factor. The addition of IS spray has been described as a practical technique to avoid HCT-based area and recovery bias in drug discovery and development [30]. 

#### 2.1.2. Capitainer^®^ B Quantitative DBS (qDBS)

The Capitainer^®^ B (Capitainer AB, Stockholm, Sweden), also known as quantitative (qDBS) or microfluidic DBS, is an example of volumetric dried blood sampling. A single drop of blood is collected and fills a capillary microchannel equipped with an inlet port. The result is a fixed volume of 10 µL of blood. Once the channel is filled, the thin film located at the inlet dissolves and the blood in excess is absorbed by an additional paper substrate (Ahlstrom grade 270), separating the excess blood. Subsequently, the thin film at the outlet is dissolved, the capillary channel is emptied thanks to capillary forces, and 10 µL of blood is absorbed on a pre-cut paper disk with a diameter of 6 mm (Ahlstrom grade 222) (Figure 2B) [31]. Two qDBSs can be collected with a single card. The device is designed to eliminate the risk of overfilling or underfilling the capillary, which also avoids sample contamination. The paper disk is protected inside the device up to the laboratory. Here, the paper disk is removed from the sample card and it is extracted for downstream analysis.

Velghe and Stove evaluated qDBS in their study on caffeine and paraxanthine and concluded that these devices effectively eliminate HCT-based bias for these two analytes. Moreover, they found that the volume added at the inlet did not impact the measured analyte concentrations [29].

#### 2.1.3. HemaSpot^TM^ HF

HemaSpot^TM^ HF (Spot On Sciences, San Francisco, CA, USA) is another quantitative sampling device for the collection of whole blood. The device contains absorbent paper (HemaForm^TM^—Spot On Sciences, San Francisco, CA, USA) with a fan-shaped design, divided into eight blades, surrounded by desiccant, inside a protective plastic box. This device is designed to integrate collection and storage since it contains an absorbent matrix and a built-in desiccant [32]. There is a protective cover on the top that allows blood collection through a central hole (Figure 2C). 2–3 drops of blood are collected and a homogeneous distribution of blood across the eight blades is promoted, allowing for repetitive and reproducible analysis while reducing the HCT effect. Additionally, it is possible to perform different downstream analyses at different times on a single sample. The total volume is ∼150 µL/device and ∼18.7 µL/blade [32].

#### 2.1.4. HemaSpot^TM^ SE

HemaSpot^TM^ SE (Spot On Sciences, San Francisco, CA, USA) is a DSS (dried serum spot) device and it is equipped with the same cartridge as the HemaSpot^TM^ HF with a similar structure. In total, 3–4 drops of blood are collected, after performing the finger-prick, on the sample collection card, which is made of a paper matrix with a spiral-shaped design. In this way, whole blood is separated into its constituents, dividing whole blood cells and serum. The separation takes about 5 min and then the cartridges are ready to be shipped for analysis, after drying for 2 min [33]. It also allows multiple punching from the same paper substrate. The device contains a spiral filter membrane that allows the separation of blood components by lateral flow, blocking cells and allowing components soluble in serum to flow directly through the form. Additionally, it features a mesh-like barrier to calibrate the rate of sample drying (Figure 2D). While red blood cells, platelets and leukocytes are concentrated in the center, serum and its components are retained in the spiral and, thus, the separation occurs (https://www.spotonsciences.com/, accessed on 13 November 2023). In lateral flow devices, a concentration gradient of analytes may be produced as a result of unrestricted flow over an undefined area. In HemaSpot^TM^ SE, cells are retained at the center while the transition to cell-free serum occurs gradually moving towards the tail of the spiral membrane [34]. Due to the chromatographic effect along the spiral, this device fails to replicate samples. HCT bias and chromatographic effect are two main drawbacks that limit analyte quantification with lateral flow devices [34].

#### 2.1.5. Telimmune Plasma Separation Card

Besides the volumetric application of DBS, there is a parallel application for dried plasma spots (DPS). Telimmune cards (Telimmune, West Lafayette, IN, USA) allow the separation of plasma from whole blood drops without centrifugation thanks to a filtration layer, using a combination of adsorption and filtration [35]. Red blood cells are trapped at the top of the separation membrane, allowing the collection of plasma that travels by capillarity [36]. In total, 1–2 drops of blood are collected on the sample card, and then plasma is separated from whole blood for approximately 3 min. Telimmune UNO delivers one DPS of 3.2 µL while Telimmune DUO delivers two identical discs of 3.2 µL of plasma. This microsampling device offers volumetric plasma collection with high reproducibility and stability at ambient temperature [37]. The collection disk is made of cellulose, has a diameter of 6.35 mm and the volume held inside is independent of the original blood volume: once the disk is saturated with plasma, the flow stops (Figure 2E). The system is not affected by factors such as HCT and blood viscosity [35]. The plasma flow is controlled by the porous membrane with a flow rate of 1 μm/s which prevents hemolysis [38]. DPS devices have been applied to toxicology, targeted metabolite quantitation and lipidomics with promising results [37]. 

### 2.2. Volumetric Tip Microsampling

Volumetric tip microsampling (VTM) is a microsampling technique to collect dried whole blood samples. The blood volume is fixed because blood is absorbed on a volumetric polymer tip and after collection, samples are dried, shipped and stored for analysis. The VTM provides HCT-independent samples, overcoming the HCT bias, uneven blood distribution and spot size variability reported with DBS traditional sampling [1]. Currently, the VTM is implemented by the commercially available devices Mitra^®^ and TASSO-M20.

#### 2.2.1. Volumetric Absorptive Microsampling

The Mitra^®^ (Trajan Scientific and Medical, Melbourne, VIC, Australia) device is based on volumetric absorptive microsampling (VAMS) technology, which was introduced in 2014 for single-drop blood collection. The collection occurs thanks to a porous hydrophilic tip that absorbs a fixed volume of blood after the finger-prick is performed on the subject. The tip is immersed and absorbs blood by wicking (Figure 2F). This device is also compatible with high throughput workflows [1] and available tips can collect volumes of 10, 20 and 30 µL with acceptable reproducibility (<4% RSD), regardless of HCT [39]. It is one of the recently developed technologies to overcome the issues of traditional DBS sampling, such as HCT bias and sample homogeneity: the porosity and quantity of polymeric material regulate the volume of blood absorbed, ensuring accurate and reproducible volumes despite HCT [40]. Denniff and Spooner assessed the effect of HCT on blood uptake by VAMS, implying ^14^C caffeine as an in vitro tracer. This study revealed that the volumetric difference in blood uptake by VAMS over a wide HCT range (20–70%) was 5%, compared with the 30% variation reported for DBS using the sub-punch method [41]. Even though VAMS is effective in overcoming the HCT bias preserving the advantages of DBS, a positive bias that led to the constant overestimation of whole blood concentrations in VAMS has been observed [42]. Various studies have also reported an impact of HCT level on analyte recovery and quantification accuracy, which could be explained by dried erythrocytes that trap analytes in the tip pores, affecting the extraction [43]. An inverse correlation was observed between accuracy and HCT level, with higher recoveries at lower HCT levels. For example, De Kesel et al. observed this trend for caffeine and paraxanthine, with lower recovery at high HCT levels [42]. This issue can be partially solved by optimizing the extraction protocol, with the selection of an appropriate extraction solvent and with the addition of a sonication step in sample preparation [43]. 

#### 2.2.2. TASSO M-20

TASSO-M20 (Tasso Inc., Seattle, WA, USA) is made of a large button that comprises a lancet, microfluidic channels and a sample collection pod with four volumetric tips (Figure 2G). The device is positioned on the upper part of the arm, the button is pressed and the lancet is released to perform the prick. The blood reaches the tips passing through the channels [1]. It delivers four samples (tips) of 17.5 µL each, with a CV < 5% (https://www.tassoinc.com/, accessed on 15 November 2023); the drying step is not required and samples can be transported right away after collection. Samples can be processed by removal of the dried blood samples from the sample pod. However, analyte extraction might be challenging due to the potential tip occlusion caused by dried erythrocyte. This issue can be overcome by pre-wetting the polymer tip with water or sonication during extraction protocol to improve the extraction efficiency [1].

### 2.3. Microneedle-Based Devices

#### 2.3.1. TAP Capillary

TAP device (YourBio Health, Medford, MA, USA) (originally Touch-Activated Phlebotomy) belongs to the category of microneedle-based devices to collect capillary blood samples through vacuum pressure [33]. The device (4.7 cm diameter, 3.5 cm height) features a stainless steel microneedle array, a pre-evacuated vacuum chamber, a microfluidic chamber and a fixed-volume container with lithium heparin anticoagulant [44]. It is positioned on the upper arm adherent to the skin, the button at the top of the device is pushed and the microneedle array is deployed and then withdrawn to start blood collection; the vacuum draws blood into the sample container and when the collection is completed, a visual indicator turns red so that the device is removed and sampling is completed in approximately 3 min (Figure 2H) [44]. Hemolysis could be produced by shear stress induced by frictional forces between blood flow and the microcapillary [33]. According to Catala et al., absolute quantitative profiles of whole blood obtained through TAP and through traditional blood sampling are comparable for a large number of classes of small molecules. Their test was based on a set of 45 targeted metabolites and they observed decreased levels of oxidative markers, suggesting decreased redox stress for TAP whole blood. According to their result, TAP microsampling seems to be a viable alternative for routine analysis of acyl-carnitines, bile acids, glycemia, lactatemia and most amino acids [45]. TAP II and TAP Micro devices (YourBio Health, Medford, MA, USA) involve the microneedle array technology for blood capillary collection and allow for the collection of up to 350 µL and up to 600 µL, respectively [46]. 

#### 2.3.2. HemaPEN^®^

HemaPEN^®^ (Trajan Scientific and Medical, Melbourne, VIC, Australia) is a volumetric HCT-independent blood microsampling device that allows the accurate collection of four whole blood samples. The device contains four micro-capillaries with a fixed volume (2.74 µL each) which provide four replicates from the same sample after the collection of a single drop of blood. Blood is collected into the four capillaries simultaneously and is absorbed into four pre-punched paper disks (3.5 mm diameter, PE 226 filter paper or 903 paper by Eastern Business Forms) [47] which areprotected from light and humidity ensuring the sample integrity (Figure 2I). HCT has no or very little impact on the volume collected by hemaPEN^®^ devices [47,48]. After collection, samples are left to dry at room temperature for 2 h and then stored at −20 °C before analysis.

## 3. Stability

Pre-analytical factors such as metabolome stability following blood collection are among the key factors to consider for microsampling devices as a potential replacement for conventional venous blood sampling. Metabolomics and lipidomics aim to characterize both the composition and amount of metabolites and lipids (metabolome and lipidome, respectively) in biological samples. Therefore, if the aim is to monitor the whole metabolome and lipidome collected by microsampling, its stability needs to be carefully evaluated. On the other hand, targeted analysis of selected metabolites and lipids requires evaluating only the stability of a few selected metabolites, lipids or drugs. In this section, we review the literature evaluating the stability of blood specimens collected by microsampling technologies. 

### 3.1. Metabolome Stability for Untargeted Metabolomics

Palmer et al. evaluated the 12-month stability of the whole metabolome in DBS stored at −20 °C, +4 °C and room temperature (+21 °C) [5]. Two untargeted ultra-high performance liquid chromatography–mass spectrometry (UHPLC-MS) approaches were applied to maximize metabolite coverage. Five time points were investigated: at 1, 14, 28 days, 6 and 12 months. It was observed that at −20 °C, DBS samples were stable within 6 months, with a change in the relative concentration of the identified metabolites of less than 1.6%. A slight increase in instability was observed between 6 and 12 months. On the other hand, the instability was greater for samples stored at 4 °C and room temperature for 6–12 months. They concluded that DBS stored at −20 °C were stable for 12 months, offering a viable tool for metabolomics studies. Moreover, 1-month storage at −20 °C and −80 °C has shown comparable levels of stability. Other studies demonstrated that short-term stability at room temperature is possible, but instability increases for long-term storage [5]. According to reported results, DBS samples can be transported and stored at room temperature for up to 28 days and then −20 °C or −80 °C is recommended for long-term storage. Long-term stability at −20 °C was evaluated for samples stored for up to 10 years to assess metabolome stability in DBS, using an untargeted metabolomics approach [23]. According to this study, the majority (>71%) of the DBS metabolome stored at −20 °C is stable for up to 10 years. The majority of the unstable metabolites were relatively unaffected by storage while only a few metabolites (e.g., methionine and glutathione) were strongly affected by storage time [23]. 

Short-term stability was investigated at −20° and room temperature for DPS (Telimmune cards) and DBS (Whatman^®^ 903) stored for 1, 7 and 28 days. It was observed that 92.2% of lipids in DPS stored at −20 °C were stable after 28 days, compared to 58.7% of lipids stable at room temperature after 28 days. Moreover, both DPS and DBS samples stored at room temperature showed that 72.7% of compounds were stable after 7 days [37]. 

Volani et al. investigated both the short- and long-term stability of VAMS and suggested that metabolome stability should be maintained for samples stored at −80 °C for up to 6 months. In contrast, samples stored at room temperature showed a significant change in their composition. In addition, metabolome stability was also affected by the time of drying: at room temperature, rapid degradation of some metabolites was observed within 48 h. According to these findings, a longer drying step has a significant impact on the sample concentration [49,50]. Short-term stability for VAMS was evaluated for samples stored at room temperature and 4 °C. The results suggested that the metabolome stability started to decrease after day 1 (variation from 10% to 25%) for samples stored in bags with desiccants at 4 °C. For longer periods of storage, a variation from 40% to 60% was observed and long-term storage must be performed at −80 °C [49]. Many studies report stability at room temperature of a wide range of analytes for prolonged times (more than 1 month) while others observed a shorter one (less than one day) for some specific compounds. The reason behind these results is associated with the intrinsic ability of specific analytes that require particular storage conditions to enhance their stability [51]. In general, to prevent deterioration due to humidity changes, VAMS devices should be stored in a firmly closed container, in the dark, and with a desiccant [51].

### 3.2. Targeted Metabolite Stability

Several studies evaluated only the stability of a few selected metabolites/lipids and/or drugs. For instance, Deprez et al. evaluated caffeine and paraxanthine stability for hemaPEN^®^ considering two different storage conditions: 4 days (short-term) at 60 °C and 2 months (long-term) at room temperature. Both metabolites were found to be stable in these two conditions [47]. In another study, Nyx et al. investigated the stability of analytes stored for 24 h at 4 °C inside the autosampler and the result was comparable to the one obtained for compounds analyzed after preparation. Long-term stability was also assessed for samples stored for 5 months at −20 °C: metabolites were stable (variation < 15%) except for 2-oxoglutaric acid, methionine, creatine, and taurine [18]. 

HemaSpot^TM^ HF’s short- and long-term stability were studied for devices stored at 4 °C, room temperature (22–25 °C) and 45 °C, at six time points (days 0, 7, 15, 30, 60, and 90). After 90 days of storage at 4 °C the antibody concentration was stable. At room temperature, it remained stable for 30 days before dropping between 60 and 90 days. At 45 °C, antibody concentration had already dropped after one day and to below 30% of the baseline after 30 days [32]. Moreover, amino acid stability for both HemaSpot^TM^ HF and SE was assessed and the results showed that phenylalanine, isoleucine, proline, valine, leucine and tyrosine were stable (showing a loss < 10%) up to 90 days up to 45 °C [52].

Marshall et al. monitored the concentration of tacrolimus and creatinine using Mitra^®^ devices. According to this study, Mitra^®^ samples were stable for 7 days at 37 °C, 14 days at room temperature and 25 days at 4 °C and –20 °C [53].

## 4. Extraction Procedure

Sample extraction from DBS devices is a critical step in the analysis process. The extraction procedure varies based on the type of sample and the yield can be impacted by variables such as temperature and storage procedure, as reported in Table 2. Therefore, sample extraction conditions must be evaluated throughout technique development and validated afterward. According to the literature, the optimal procedure for extracting DBS samples is to use methanol and water as solvents. 

### 4.1. Extraction of Untargeted Metabolites

Palmer et al. studied the 12-month stability of DBS samples that were extracted with 100 µL methanol and water in a ratio of 4:1 (*v*/*v*). Although the procedure yielded optimal results, it was demonstrated that analyte concentrations declined when methanol was employed as an extraction solvent, probably due to continual drying and absorption onto the filter paper over the course of a year. Additional research is required to support this evidence [5].

Marasca et al. tested different extraction solvents for the untargeted analysis of the whole blood lipidome. They investigated four extraction solvents (isopropanol, methanol, methanol/chloroform and methanol/tert-butyl methyl ether) for liquid blood, DBS and VAMS. The optimal extraction conditions were chosen to take into account the total number of lipid species identified, which led to the following results: 100% isopropanol (IPA) for liquid blood, 50:50 methanol/tert-butyl methyl ether (MeOH/MBTE) (*v*/*v*) for VAMS and 90:10 methanol/chloroform (MeOH/CHCl_3_) (*v*/*v*) for DBS. According to this study, 100% MeOH is more efficient in extracting free cholesterol and sphingolipids, while MeOH/MBTE is more suitable for less polar lipids (e.g., TAG, CE) when working with DBS. Both MeOH/CHCl_3_ and MeOH/MTBE are efficient in extracting a variety of lipid classes (sphingolipids, free cholesterol, free fatty acids, phospholipids) from VAMS. For broad lipid class profiling, VAMS seems to be more flexible and efficient overall [54].

The main disadvantage of DBS samples is that the analysis is impaired by blood cell components. Kim et al. investigated the advantages of sample collection on paper as a dried plasma spot (DPS). The collection disc was dried for 15 min, and 2 μL of mixed IS in methanol was added to the collection disc and dried again. Extraction was performed by pipetting 20 μL of methanol directly onto the disc [35]. The reproducibility of volume sampling in DPS was evaluated by Johnson et al. using data from ten extraction cards using blood from the same participant. Metabolites for global profiling are extracted using 500 µL of 100% MeOH vortexed briefly. In addition, 250 µL of chloroform and 500 µL of ultra-pure, filtered water are added later in the extraction process. The obtained results demonstrate that the Telimmune Plasma Separation Card (TPSC) is capable of reproducible sample collection that is independent of applied blood volume or hematocrit [55].

A mixture of acetonitrile and water is a potential extraction solution for hemaPEN^®^ devices. According to Nix et al., the fundamental benefit of microsampling with hemaPEN^®^ consists of the fact that sampling can be carried out efficiently and conveniently at the training site [9]. Although numerous studies demonstrating metabolomic changes during or after physical activity have already been published [56], none allowed athletes to collect blood by themselves at the training site [18].

### 4.2. Extraction of Targeted Metabolites

Lee et al. tested three extraction solvents—methanol, acetonitrile, methanol/acetonitrile in a ratio of 1:1 (*v*/*v*), in volumes ranging from 20 µL to 200 µL. As the results highlighted, all the target compounds were well extracted with acetonitrile. However, using methanol as an extraction solvent, the extraction efficiency was higher [47]. Despite the fact that some metabolites could be extracted more effectively with methanol than with a methanol/acetonitrile mixture, the differences were inconsequential [57]. Li et al. also employed a mixture for analyte extraction. The buffer was a 40:40:20 *v/v* solution of acetonitrile, water, and methanol. Different extraction volumes ranging from 100 to 500 µL were investigated in order to optimize the methodology. In terms of the extraction’s yield, there were no discernible changes across the different volumetric approaches [58]; Beck et al. collected DBS samples on standard filter paper that were subsequently placed in a test tube, and then 10 μL of IS solution and 125 μL of methanol were added. The value of the volumetric DBS measurement of the analyte was examined using a disposable prototype DBS device. The volumetric filter disc was extracted with 200 µL isopropanol containing IS [59].

The hemaPEN^®^ device was utilized by Deperez et al. as an alternative HCT-independent DBS device. The extraction method used 70 µL methanol:water (80:20, *v*/*v*) + 0.01% formic acid. The absence of extractability difficulties when working with authentic samples was determined by storing hemaPEN^®^ devices for 4 days at 60 °C after drying at room temperature [47]. Methanol and water were used also by Laurent et al. In this study, four different extraction solvents were investigated: methanol:water (80:20, *v*/*v*), ethanol:water (80:20, *v*/*v*), methanol:methyl-tert-butyl ether (50:50, *v*/*v*), and ethanol:methyl-tert-butyl ether (50:50, *v*/*v*). Two separate approaches were used to test each extraction solvent and the best recovery was obtained by utilizing the first described solutions as the extraction solvent in the second protocol [48].

Volumetric absorptive microsampling (VAMS) allows for the collection of precise and accurate blood volumes, overcoming the HCT impact associated with DBS. To optimize the extraction procedure, Paniagua-González et al. tested different extraction solvents, including methanol, acetonitrile and methanol:water (80:20, v:v). Outcomes obtained in each case were compared and the best results in terms of sensitivity were observed with methanol:water (80:20, v:v) as previously found by Koster et al. [60]. However, the best results overall were obtained by sonicating the VAMS samples with methanol:water (40:60, v:v) for 15 min, and sonicating for 15 more min after the addition of 200 μL methanol (methanol:water, 80:20, v:v) [61]. In another study, Denniff et al. extracted the tips of dried VAMS samples with 200 μL of methanol containing internal standard (2.5 μg/mL) and 0.02 mg of EDTA per tip. In this case, EDTA was added to enable comparisons with levels obtained using standard blood collection tubes [62].

**Table 2:** Methods of extraction. Acetonitrile: ACN; chloroform: CHCl_3_; isopropanol: IPA; lithium chloride: LiCl; methanol: MeOH; tert-butyl methyl ether: MTBE.

Type	Sample	Extraction	Source
DBS	whole blood	125 µL MeOH 200 µL IPA	[59]
DBS	whole blood	225 µL MeOH:H_2_O (80:20, *v*/*v*) + 0.01% FA	[29]
DBS	whole blood plasma	100 µL MeOH:H_2_O (4:1, *v*/*v*) 80 µL MeOH	[5]
DBS	whole blood	100 µL MeOH (80%)	[23]
DBS	whole blood	450 µL MeOH + 150 MTBE	[63]
DBS	whole blood	20 µL, 50 µL, 200 µL MeOH 20 µL, 50 µL, 200 µL ACN 20 µL, 50 µL, 200 µL MeOH/ACN (1:1 *v*/*v*)	[57]
DBS	whole blood	40 µL H_2_O + 160 µL MeOH	[3]
DBS	whole blood	225 µL MeOH/H_2_O (80:20 *v*/*v*) + 0.01% FA	[29]
DBS	whole blood plasma	200 µL MeOH:ACN:H_2_O (40:40:20, *v*/*v*/*v*)	[58]
DBS	whole blood	4 mL CHCl_3_/MeOH (1:1 *v*/*v*) + 1.6 mL LiCl solution (50 mM)	[64]
DBS	whole blood	MeOH/CHCl_3_ (90:10 *v*/*v*)	[54]
hemaPEN^®^	whole blood	ACN:H_2_O (60:40 *v*/*v*)	[18]
hemaPEN^®^	whole blood	MeOH:H_2_O (80:20, *v*/*v*)	[48]
hemaPEN^®^	whole blood	70 µL MeOH:H_2_O (80:20, *v*/*v*) + 0,01% formic acid	[47]
VAMS	whole blood	MeOH:H_2_O (80:20, *v*:*v*) MeOH:H_2_O (60:40, *v*:*v*)	[61]
VAMS	whole blood	200 µL MeOH	[62]
VAMS	whole blood	MeOH/MBTE (50:50 *v*/*v*)	[54]
TELIMMUNE	plasma	(1) 500 µL MeOH (2) 250 µL of CHCl_3_ (3) 500 µL of ultra-pure filtered water	[55]
TELIMMUNE	plasma	500 µL MeOH: H_2_O (50:50, *v*:*v*)	[36]
TELIMMUNE	plasma	20 µL MeOH	[35]
TELIMMUNE	plasma	25 µL of phosphate-buffered saline (pH 7.4)	[65]

## 5. Microsampling Applications Monitoring Lipids and Metabolites

Due to its numerous advantages, microsampling technology has become widely utilized in blood collection and storage across various fields [25]. In recent years, there has been a notable increase in studies combining microsampling with mass spectrometry techniques [1,21,66,67], with mass spectrometry being the preferred analytical tool for measuring lipids and metabolites. The synergy of microsampling with mass spectrometry brings forth several benefits, including minimal sample requirements, the ability to analyze multiple analytes simultaneously, and high sensitivity and specificity [1,67]. 

As mentioned earlier, microsampling can be carried out by the patient without requiring trained personnel. This feature simplifies remote sampling, enhancing accessibility for individuals in remote areas and eliminating unnecessary, and in some cases, risky hospital visits for elderly individuals [68]. Moreover, due to its non-invasive nature, microsampling facilitates longitudinal studies [69]. Below, we provide an overview of some major applications of microsampling for monitoring lipids and metabolites.

### 5.1. Population Studies and Newborn Screening

Population studies can also take advantage of DBS technologies both for remote sampling with self-collection of blood and longitudinal studies. One of the main applications of DBS in population studies is newborn screening, which is nowadays used as a screening approach on all newborns for diagnosis of specific congenital disorders and conditions whose late diagnosis could result in the onset of irreversible symptoms [70]. NBS was first introduced in Europe in the 1960s for the screening of phenylketonuria, a metabolic disorder that affects the body’s ability to process amino acid phenylalanine [71]. Over the last decades, the development and the introduction of tandem mass spectrometry enlarged the panel of screened diseases (or conditions) that can be analyzed [72], making possible the screening for 40–50 conditions using a single blood spot [73]. In most western countries, a combined legislative scheme provided a nationwide NBS for over 40 disorders [74]. The screening is usually performed using a triple quadrupole mass spectrometer with a targeted metabolomics approach able to quantify amino acids and acylcarnitines [75]. 

Numerous studies conducted in recent years proposed new potential biomarkers for newborns’ diseases. The work of Mak et al. allowed the identification of a metabolic panel of 121 metabolites using DBS collected from newborns with an untargeted metabolomics approach. This panel could be used in second-tier assays to reduce the number of false positives for four metabolic diseases, glutaric acidemia type I (GA1), methylmalonic acidemia (MMA), ornithine transcarbamylase deficiency (OTCD), and very long-chain acyl-CoA dehydrogenase deficiency (VLCADD) [76]. Moreover, Brown and colleagues used tandem mass-spectrometry to identify short-chain carnitine and ornithine as potential biomarkers in newborns of succinic semialdehyde dehydrogenase deficiency (SSADHD) [77], a rare genetic disease associated with tissue accumulation of γ-aminobutyric acid and γ-hydroxybutyric acid neuromodulators, and tissue depletion of glutamine and glutamic acid [78]. 

In a very recent study, newborn DBS metabolomics has been used to explore the correlation between prenatal per- and polyfluoroalkyl (PFAS) exposure and gestational age at birth outcomes, finding that maternal serum PFAS levels during early to middle pregnancy, are associated with early birth prior to full-term among African American pregnant people and their newborns [79].

Another potential application of DBS is represented by disease surveillance, which is a valuable tool in epidemiology and public health. In fact, it is critical to monitor and control the spread of diseases, respond to outbreaks and inform healthcare interventions in specific populations, especially in resource-constrained or remote areas with difficult access to hospitals. Templer and colleagues indicate a new DBS device for blood collection with increased sensitivity for real-time PCR analyses [80]. This could lead to lower HIV-related morbidity and death as well as early detection and treatment of newborns infected with the virus [80]. At the moment, to the best of our knowledge, few publications have combined microsampling with metabolomics or lipidomics-based epidemiology studies, but the introduction of DBS devices and their advantageous properties could allow these kinds of studies also in rural areas. Indeed, self-sampling has the potential to increase the number of participants in large-scale studies. Even though evaluation or preanalytical issues related to self-sampling, variations in collected volume and hemolysis have been poorly evaluated and tested [81].

### 5.2. Nutritional Studies 

Another field that might be interested in using microsampling and self-sampling strategies is nutritional metabolomics, which has the goal of correlating dietary patterns with health.

Petrick et al. investigated the impact of early life exposure on pediatric acute lymphoblastic leukemia (ALL) by conducting an untargeted metabolomics study. Neonatal DBS were collected within days from birth and analyzed via liquid chromatography high-resolution mass spectrometry (LC-HRMS). Since the age at diagnosis is an influencing factor, patients were grouped by early (1–5 years) and late (6–14 years) diagnosis. Results showed that levels of linoleic acid and linolenic acid were higher in the late-diagnosis group. Moreover, these two fatty acids were found in greater concentrations in children who were fed formula instead of breast milk, suggesting that nutrition in the early stages of life may be correlated with risks of ALL [82].

In the work of Pfluger and colleagues, DBS devices were used to find novel nutrition and metabolic indicators during infant weaning in Malian infants from 6 to 12 months supplied with heat-stabilized rice bran. The untargeted metabolomics analysis pointed out that heat-stabilized rice bran represented a great source of nutrients during weaning especially in low- and middle-income regions where nutritional deficiencies and nutrient deficiency and food scarcity are frequent. The supplementation leads to an increased concentration of several metabolites which are involved in multiple metabolic processes, including antioxidant defenses (reduced glutathione, glutamate and glycine), lipid profiles (short and medium-chain fatty acids), and neuroactive pathways (glutamic and aspartic acid, glycine, and asparagine) [83].

McNairn et al. investigated food intake by postprandial DBS collection in eight healthy volunteers. The aim of the study was to distinguish a high-fat, high-protein meat (HFPM) diet from a high-carbohydrate vegan (HCV) diet using metabolomics analysis. Blood samples were taken 3 h after breakfast and after lunch. In both postprandial DBS, higher levels of acylcarnitines, creatine, cis-trans-hydroxyproline and triacylglycerols were found in the HFPM diet. On the other hand, the HCV diet led to higher sorbitol concentrations. In summary, the two diets resulted in two significantly different metabolomics profiles in DBS. This study demonstrates that dietary metabolomics in DBS was able to distinguish the HFPM and HCV diets and can be an effective approach to monitoring food intake. It may be a useful tool to provide a more objective measure of food intake and enable a complementary alternative to conventional dietary assessment procedures [84]. 

### 5.3. Drug Discovery

Microsampling approaches have the potential to be integrated at various stages of drug development studies. Even though few studies have been published on this topic, microsampling has been probably used during metabolomics studies by the pharmaceutical industries in phase 1 and phase 2 of drug development, as evidenced by data compiled from responses received from 39 pharmaceutical companies and contract research organizations [85]. A substantial number of respondents have submitted microsampling data, both from nonclinical and clinical studies, to various regulatory agencies [85]. Notably, microsampling finds more routine adoption in nonclinical studies compared to clinical studies. Within nonclinical investigations, microsampling approaches are increasingly prevalent in discovery phase projects as opposed to later-phase non-GLP and GLP studies [85]. 

Therapeutic drug monitoring (TDM) is a specialized clinical practice that consists of the measurement of the concentration in plasma or blood of a wide range of drug classes, such as antiepileptics, antimicrobial agents and immunosuppressants [86,87,88,89,90,91]. Most of the studies are focused on the quantification of the drugs in blood or serum using DBS [92], but in recent years, pharmacometabolomics has emerged, aiming at the evaluation of the metabolic fingerprints following drug administration [93]. Even with some limitations, i.e., the standard protocol for sample preparation and storage, environment influence, and ethnicity, this type of study could be useful to evaluate drug toxicity and pharmacokinetics, but also predict the pharmaceutical answer and its pharmacodynamics [94].

One of the main advantages of microsampling techniques is that they can be easily extended in studies where animal models are included [95,96,97]. The use of this technique represents an important and useful tool for blood sampling during the study, and in accordance with two out of three of the 3Rs, reduction and refinement. In fact, microsampling devices allow multiple sampling, without pain and stress in the animal during the collection, and consequently permit the reduction in the number of animals necessary for the study [96]. For instance, Volani et al. demonstrated that the integration of VAMS technology and MS-based metabolomics allows one to find specific longitudinal metabolic variations after iron supplementation [95].

### 5.4. Microsampling in Sport

In the realm of sports, the integration of metabolomic blood analyses and microsampling provides a unique avenue to delve into the molecular repercussions of physical activity. In this context, multiple sampling is often required and often blood collection is performed during competition of training in the field. Therefore, microsampling simplifies the logistics of sample collection and facilitates the possibility of using omics analysis in evaluating dynamic changes in the metabolome during physical activity.

Puigarnau et al. delved into the impact of high-intensity activities, such as trail running, on blood metabolites. Employing volumetric absorptive microsampling devices (Mitra^®^ Clam-shell, Neoteryx, Trajan Scientific and Medical, Melbourne, VIC, Australia), capillary blood was collected from thirty-three participants before and after the race. Stratifying participants based on training levels—low, moderate, and high—revealed significant variations in pre and post-race concentrations of metabolites like taurine and acetyl-carnitine. Notably, highly trained athletes exhibited minimal alterations compared to their less-trained counterparts [98].

Cendali et al. conducted a parallel study, focusing on running exercise. Blood samples from twelve volunteers were collected before, immediately after, and 24 h post-activity. Mass spectrometry analysis highlighted the DBS approach’s potential for longitudinal metabolic profiling, revealing sustained changes in nitrogen homeostasis-related compounds. Gender-specific variations were unraveled, potentially linked to the chromosome X location of the glucose 6-phosphate dehydrogenase (G6DP) gene, with consequential impacts on lactate and pyruvate levels [99].

Nix et al. pursued a targeted metabolomics analysis, utilizing microsampling with hemaPEN^®^ devices (Trajan Scientific and Medical, Melbourne, VIC, Australia). Twenty participants underwent a 400 m warm-up followed by acute running exercise, monitored at multiple time-points. Lactic acid emerged as a pivotal metabolite, exhibiting a significant surge during exercise, reverting to baseline levels half an hour post-exercise, aligning with the existing literature trends [18]. 

Nemkov et al. characterized molecular exertion profiles in elite athletes during a World Tour cycling competition. Untargeted metabolomics and lipidomics analysis were performed on blood samples from 28 elite male athletes using VAMS devices. The blood samples were collected before and after a graded exercise test and before and after a long aerobic training. Furthermore, five cyclists were selected for additional analysis during a seven-stage elite World Tour race. The graded exercise test highlighted a significant accumulation of lactate, succinate, free fatty acids and acylcarnitines. During the long aerobic exercise session, a significant increase in fatty acids and acylcarnitines was observed without major changes in lactate and succinate. A similar result was obtained in samples from sprinting and climbing stages of the World Tour, along with an increased fatty acid oxidation capacity associated with competitive performance. This study provides a useful insight into the metabolomic and lipidomic changes in blood during elite competition. It also demonstrates the benefits of microsampling devices that allow blood sampling in training and in competition [100].

Bassini et al. investigated the inflammatory response to understand exercise-induced changes by combining sportomics with DBS and multiplex mass spectrometry. Since exercise is known to lead to changes in acute-phase proteins that may be associated with overtraining, a panel of 11 blood proteins in 687 samples was analyzed. Blood samples were collected using Whatman 903 Protein Saver cards from 97 elite and Olympic-level athletes (men and women) from 16 sports. The results show that five acute phase proteins were highly correlated and that this correlation varied among the 16 sports analyzed. For example, CRP-SAA1 (C-reactive protein-serum amyloid A1) and CRP-LBP (C-reactive protein-lipopolysaccharide-binding protein) were found to be highly correlated in acute inflammation in response to exercise. In addition, a correlation was suggested between the reduction in TLR4 (Toll-like receptor 4) and the protective effect of exercise in heart diseases. A total of 1500 protein–protein interactions were highlighted in this study and 30 of the 44 core proteins were associated with immune system processing [101].

### 5.5. Multi-Omics and Microsampling

The emerging field of multi-omics refers to the integrative analysis of various biological data sets, including genomics, transcriptomics, proteomics, metabolomics, and epigenomics. Using several layers of molecular data at once, this method seeks to give a thorough and all-encompassing knowledge of complex biological systems and opens a new horizon for precision medicine [102]. Such an integrative approach has been used on entire blood and plasma, achieving promising results in the understanding of complex biological processes, leading to more precise and personalized diagnostics as well as targeted therapeutic interventions. As such, multi-omics approaches allowed us to improve the characterization of COVID-19 infection, identifying both predictive biomarkers of severity [103] and anti-inflammatory response [104]. A similar strategy has been applied to investigate possible biomarkers of neurological diseases [105,106,107] and metabolic disorders [108,109,110]. However, the initial stage of sample collection and handling still represents a bottleneck for large-cohort and long-term studies [111]. In this context, a microsampling approach could play a key role in minimizing the invasiveness of sample collection and the consequent stress for the subjects under investigation. Interestingly, Shen et al. [111] performed two case studies involving participants in their native environments. The first case study aimed to investigate the effects of drinking a complex mixture on metabolic profiles at different timepoints. The study included 32 participants who were mailed the microsampling kits and the same shake. The subjects were instructed to collect the first sample prior to consuming the shake, as well as four additional samples at 30, 60, 120, and 240 min after consumption. Microsamples were then used to extract metabolomics, lipidomics, and cytokines/hormones profiles. The multi-omics results revealed different alterations in time, reporting significant alterations among different classes of analytes at different time points. Specifically, the changes in molecules were classified into three major clusters across five time points. The first cluster consisted primarily of amino acids and cytokines, which increased rapidly with a peak at 60 min and then decreased. The second cluster contained mostly lipids increasing up to 60–120 min before decreasing. The third cluster included mostly acylcarnitines, decreasing in response to shake consumption and recovering after 240 min. Additionally, the t-distributed stochastic neighbor embedding (tSNE) plot of the multi-omic data revealed that each participant had a unique molecular profile that could be further classified into two main groups depending on their ability to respond to the shake. Furthermore, the authors investigated the kinetics of each class of metabolite, defining six metabolic scores: carbohydrate, lipid, free fatty acids (FFAs), protein, and insulin secretion cytokines. Thus, the multi-omics data from microsamples revealed significant heterogeneity in the biochemical responses of each individual to a complex nutritional complex. In the future, such data could be linked to medical phenotypes and used to provide personalized nutrition management. The second case study proposed by Shen et al. focused on a 24/7 personalized whole physiology profiling of a single individual using wearable and multi-omics data. Over the course of 7 days, a single participant collected blood microsamples every 1–2 h during waking hours, collecting a total of 98 microsamples. On the same days, a smartwatch was used to record heart rate (HR) and step count, along with a continuous glucose monitor (CGM) and food logging was also recorded. In-depth multi-omics profiling, including untargeted proteomics, untargeted metabolomics, targeted lipidomics and targeted cytokine, hormone, total protein, and cortisol assays, was performed on the 98 microsamples. A total of 2213 analytes were annotated among metabolites, lipids, and proteins. To examine if the measured metabolic changes in the individual could reflect real changes, the authors verified metabolic changes in a day with high carbohydrate consumption and in a day with low carbohydrate intake, confirming the validity of the test. It was also possible to monitor cortisol levels changes across the day, following not only circadian rhythms but also stress levels. The frequent sampling also allowed monitoring of inflammatory events, corresponding to an increase in cytokines occurring without symptoms. These data in particular could be of extreme importance for monitoring patients and for the early detection of disease. Correlating the multi-omics with the wearable data showed a correlation between specific molecular classes and short-term physiological changes. Overall, the results of this pilot study provide a valuable basis for further multi-omic investigations with longitudinal studies. In this context, microsampling brings important advantages when aiming at personalized medicine. The low amount of sample and the low invasiveness of the procedure make it ideal for high-frequency collection and longitudinal biomarkers [112]. Nonetheless, the investigation of other molecular levels and the investigation of large sample cohorts will certainly require improvements in data analysis and integration. Furthermore, future multi-omics studies must consider the limitations of traditional DBS, such as hematocrit volume and analyte stability, self-sampling, and hemolysis. Moreover, proteins, RNA, DNA, and/or glycans, might require specific extraction and storage conditions. However, these limitations may be overcome by technological advances in both DBS systems and optimization of the analytical and preanalytical aspects [113]. 

## 6. Conclusions and Future Directions

The widespread adoption of microsampling technology in blood collection has revolutionized various fields, driven by its numerous advantages. The integration of microsampling with mass spectrometry techniques, especially for lipids and metabolite analysis, has ushered in a new era with benefits such as minimal sample requirements, simultaneous analysis of multiple analytes, and enhanced sensitivity and specificity.

The capacity for patients to perform microsampling without the need for trained personnel simplifies remote sampling, making it accessible for individuals in remote areas and eliminating unnecessary and, at times, risky hospital visits for the elderly. Additionally, the non-invasive nature of microsampling facilitates longitudinal studies, allowing for the monitoring of dynamic changes in metabolic profiles over time.

Exploring major applications, microsampling for the analysis of lipids and metabolites has proven valuable in population studies, particularly in newborn screening for congenital disorders. The technology’s potential spans wellness and disease surveillance, nutritional studies, sports medicine, and drug discovery. Furthermore, the emerging field of multi-omics, which integrates genomics, transcriptomics, proteomics, metabolomics, and epigenomics, presents a promising avenue for precision medicine.

As with any new analytical platform, the implementation of microsampling technologies for the analysis of lipids and metabolites will require standardization of both pre-analytical and analytical strategy protocols. The field continues to innovate with the introduction of new microsampling devices, promising further advancements in biomedical research. In the future, as microsampling becomes more prevalent, continued advancements in data analysis and integration will be essential to unlock the full potential of this technology, particularly in large-scale and long-term studies. Overall, microsampling stands at the forefront of transformative approaches in biomedical research, offering unparalleled opportunities for precision medicine and in-depth exploration of complex biological systems.

## Figures and Tables

**Table 1:** Overview of microsampling. In the table are listed the type of sample and microsampling, the device and the manufacturing company, if it is volumetric, the sample volume, the potential HCT bias and collection for each device. The average time of sampling for each device is around 2–3 min.

Device	Company	Sample Type	Collection	Volume	Volumetric	Potential HCT Bias
Whatman^®^ 903	Cytiva, Global	Dry whole blood	Capillary blood after finger-prick	20–80 µL	✘	✔
Capitainer^®^ B	Capitainer AB	Dry whole blood	Capillary blood after finger-prick	10 µL	✔	✘
HemaSpot^TM^HF	Spot On Sciences	Dry whole blood	Capillary blood after finger-prick	~18.7 µL/blade ~150 µL/device	✔	✘
HemaSpot^TM^ SE	Spot On Sciences	Dry serum	Capillary blood after finger-prick	~150 µL/device	✔	✔
Telimmune UNO/DUO	Telimmune	Dry plasma	Capillary blood after finger-prick	3.2 µL	✔	✘
Mitra^®^	Trajan Scientific	Dry whole blood	Capillary blood after finger-prick	10, 20, 30 µL	✔	✘
TASSO M-20	Tasso INC.	Dry whole blood	Capillary blood from the upper arm. Push-button device	17.5 µL	✔	✘
TAP II TAP Micro	YourBio Health	Liquid whole blood	Capillary blood from the upper arm. Push-button device	up to 350 µL up to 600 µL	✔	✘
hemaPEN^®^	Trajan Scientific	Dry whole blood	Capillary blood after finger-prick	2.74 µL 10.96 µL/device	✔	✘

✘: the microsampling device is not volumetric/does not show potential HCT bias; ✔: the microsampling device is volumetric/shows potential HCT bias.
